# The Immunodominance Change and Protection of CD4^+^ T-Cell Responses Elicited by an Envelope Protein Domain III-Based Tetravalent Dengue Vaccine in Mice

**DOI:** 10.1371/journal.pone.0145717

**Published:** 2015-12-29

**Authors:** Hsin-Wei Chen, Hui-Mei Hu, Szu-Hsien Wu, Chen-Yi Chiang, Yu-Ju Hsiao, Chia-Kai Wu, Chun-Hsiang Hsieh, Han-Hsuan Chung, Pele Chong, Chih-Hsiang Leng, Chien-Hsiung Pan

**Affiliations:** 1 National Institute of Infectious Diseases and Vaccinology, National Health Research Institutes, Miaoli, Taiwan; 2 Graduate Institute of Immunology, China Medical University, Taichung, Taiwan; University of Iowa, UNITED STATES

## Abstract

Dengue is the leading cause of mosquito-borne viral infections and no vaccine is available now. Envelope protein domain III (ED3) is the major target for the binding of dengue virus neutralizing antibodies; however, the ED3-specifc T-cell response is less well understood. To investigate the T-cell responses to four serotypes of dengue virus (DENV-1 to 4), we immunized mice using either a tetravalent ED3-based DNA or protein vaccine, or combined both as a DNA prime-protein boost strategy (prime-boost). A significant serotype-dependent IFN-γ or IL-4 response was observed in mice immunized with either the DNA or protein vaccine. The IFN-γ response was dominant to DENV-1 to 3, whereas the IL-4 response was dominant to DENV-4. Although the similar IgG titers for the four serotypes were observed in mice immunized with the tetravalent vaccines, the neutralizing antibody titers varied and followed the order of 2 = 3>1>4. Interestingly, the lower IFN-γ response to DENV-4 is attributable to the immunodominance change between two CD4^+^ T-cell epitopes; one T-cell epitope located at E_349-363_ of DENV-1 to 3 was more immunogenic than the DENV-4 epitope E_313-327_. Despite DENV-4 specific IFN-γ responses were suppressed by immunodominance change, either DENV-4-specific IFN-γ or neutralizing antibody responses were still recalled after DENV-4 challenge and contributed to virus clearance. Immunization with the prime-boost elicited both IFN-γ and neutralizing antibody responses and provided better protection than either DNA or protein immunization. Our findings shed light on how ED3-based tetravalent dengue vaccines sharpen host CD4 T-cell responses and contribute to protection against dengue virus.

## Introduction

Dengue is the most prevalent mosquito-borne infectious disease and has spread to over 100 countries due to global warming and an increase in international travel [1]. It is estimated that 400–500 million dengue infections occur annually and that one quarter of these cases are symptomatic, resulting in 21,000 deaths per year [2]. In addition to vector control, a reliable preventive dengue vaccine is needed more urgently than ever to reduce the threat of dengue. However, the complexity of interactions between the four serotypes of dengue virus (DENV-1 to 4) and the poorly understood mechanisms of immune protection impede the development of a dengue vaccine [3]. After primary dengue infection, both serotype-specific/homotypic and cross-reactive/heterotypic immune responses are elicited. However, due to the lack of long-lasting cross-protection, the heterotypic immune responses have been reported to be less protective and associated with severe dengue diseases, including dengue hemorrhagic fever and dengue shock syndrome [4]. For example, antibody-dependent enhancement (ADE) and the concept of original antigenic sin mediated by cross-reactive antibodies and T cells have been proposed in the pathogenesis of severe dengue [5, 6]. Therefore, it is believed that an ideal dengue vaccine would be able to induce balanced immunity against all dengue serotypes.

Neutralization is well known to play an important role in blocking dengue virus infection. Although all exterior viral proteins theoretically can induce neutralizing antibodies, domain III of the dengue envelope protein (ED3) has been reported to be the major target for serotype-specific neutralizing antibodies [7, 8]. In addition, immunization with DNA encoding ED3 or recombinant ED3 subunits has been shown to induce protective antibodies against dengue virus in mouse and non-human primate models [9–11] and to reduce the risk of ADE [12]. However, ED3 is not as immunogenic as the entire envelope protein [13]; therefore, some enhancements are required for ED3-based dengue vaccines, including the addition of a signal peptide for secretion [13] or other dengue proteins containing T-cell epitopes [14, 15] and the use of an adjuvant.

CD4^+^ T-cell responses are very important for antibody responses. However, although numerous studies have focused on neutralizing antibody epitopes, the role of ED3-specific CD4^+^ T-cell responses has been less thoroughly investigated, and most identified CD4^+^ T-cell epitopes have focused on DENV-2 [16–18]. Considering that four serotypes antigens with high amino acid sequence homologies co-exist in hosts that received a tetravalent dengue vaccine, the T-cell responses to different serotypes will be more complicated. For example, the different amino acids in a T-cell epitope (or altered peptide ligand) will affect the affinity between TCR and the MHC-peptide complex and determine whether the T-cell response is serotype-dependent or cross-reactive [19, 20]. In addition, more evidences from human and animal studies indicates that IFN-γ-producing T cells contribute to protection against dengue virus [21–23]; these findings highlight the importance of a systematic analysis of the IFN-γ-producing T-cell responses after multivalent dengue vaccination.

In this study, we used a tetravalent ED3-expressing DNA vaccine and a tetravalent recombinant ED3 subunit vaccine formulated with an alum adjuvant, as well as the combination of both as a DNA prime-protein boost vaccination, to investigate the ED3-specific CD4^+^ T-cell response; we then evaluated the protection of this response against dengue virus challenge in a mouse model.

## Materials and Methods

### Ethics statement

Animals were obtained from the National Laboratory Animal Center (Taipei, Taiwan) and were maintained in the animal facility of the National Health Research Institutes. The protocol was approved by the Animal Committee of the National Health Research Institutes (Protocol No: NHRI-IACUC-103067-A) and was performed according to their guidelines. For the care and use of animals utilized in this research, we monitored the animals twice per week and none of animals showed severe ill, died or moribund required for humane endpoints during the whole experiments. A protocol for early euthanasia/humane endpoints is performed if one of the following criteria is met: the loss of body weight more than 20%, a wound that cannot be improved after medication or animals developing neurological symptoms and unable to feed by themselves. For anesthesia and euthanasia/ humane endpoints, mice were treated with 2–3% of isoflurane and 3% of CO2 inhalation, respectively.

### Cloning of the dengue DNA vaccine

The consensus amino acid sequence for the ED3 (E_295-397_) of the four dengue virus serotypes has been described previously [24]. For the tetravalent ED3 DNA vaccine, we designed a mixture of two plasmids, which contained tandem repeats of bivalent ED3 from DENV-1 and 3 (pDV13-ED3) or DENV-2 and 4 (pDV24-ED3), separated by a linker (three repeats of Gly-Gly-Gly-Gly-Ser; GGGGS x 3). The codon-optimized cDNA fragments encoding bivalent ED3 were synthesized (Genscript, Piscataway, NJ, USA) and inserted into the pVax-1 expression vector between Xho I and Apa I sites. A leading signal peptide from immunoglobulin light chain (METDTLLLWVLLLWVPGSTGD) was inserted onto the N-terminus of bivalent ED3 to direct the expressed protein to the secretion pathway.

### Production of recombinant ED3 protein

The preparation of dengue ED3 protein was performed as previously described [12]. Briefly, four serotypes of consensus ED3 cDNA were synthesized and cloned into the pET-22b (+) vector and expressed in *Escherichia coli* BL21. Recombinant ED3 was purified by immobilized metal affinity chromatography. The eluent from the affinity column was then polished using an anion exchange column (DEAE sepharose fast flow; GE) after dialysis against DEAE buffer [50 mM NaH_2_PO_4_/1 M urea (pH 5.8)]. An E membrane (Pall, USA) was used to remove endotoxin. Endotoxin levels of purified ED3 were determined using the Limulus amebocyte lysate (LAL) assay (Associates of Cape Cod, Inc. Cape Cod, MA), and the resulting endotoxin levels were less than 0.06 EU/mg.

### Cell transfection and Western blotting

The expression of pDV13-ED3 and pDV24-ED3 was tested by *in vitro* transfection. Briefly, 5 μg of plasmid DNA was diluted into 200 μl of DMEM and mixed with 10 μl of Lipofectamin 2000 (Invitrogen) at room temperature (RT). Following a 20-min incubation, the DNA- Lipofectamin mixture was slowly added into 80% confluent 293Trex cells (Invitrogen) and filled with complete DMEM medium containing 10% FBS to a final volume of 2 ml 4h later. After 24 h of incubation, the culture supernatant and cell lysate were separately collected and stored at -20°C. The samples containing 1 μg of total cellular protein or 30 μl of culture supernatant were loaded into the wells of a 4–20% gradient SDS-PAGE for electrophoresis. For Western blotting, proteins in the gel were transferred onto a nitrocellulose membrane and blotted with a monoclonal anti-dengue antibody (GeneTex) or a monoclonal antibody against β-actin followed by a horseradish peroxidase (HRP)-conjugated goat anti-mouse IgG antibody (Pharmacia) and then developed by adding the substrate. For purified recombinant ED3, 0.5 μg of total protein was used, and the process was performed as described above, except that the detection antibody was replaced with hyperimmune sera from mice immunized with tetravalent DNA vaccine and boosted with tetravalent recombinant ED3 protein.

### Immunization and challenge

Groups of BALB/c mice (6–8 weeks of age) were immunized subcutaneously with tetravalent recombinant ED3 (10 μg for each serotype) plus alum as an adjuvant or with tetravalent DNA vaccine (pDV13-ED3 and pDV24-ED3; 100 μg each) or vector control pVax-1 (200 μg) by intramuscular injection. Mice were given two boosts at two-week intervals with either homologous or heterologous vaccines. Mice received a challenge 4 weeks after the last immunization via subcutaneous injection of 5 x 10^7^ K562 cells that had been infected with DENV-4/H241, as published elsewhere [25].

### Enzyme-linked immunospot (ELISPOT) assay

Production of IFN-γ and IL-4 by mouse spleen cells was measured using an ELISPOT assay, as described elsewhere [26]. In brief, multiscreen plates (Millipore) were coated with 2 μg/ml of anti-mouse IFN-γ or anti-mouse IL-4 antibody (all from BD Pharmingen). After the plates were washed and blocked with culture medium, 1x10^5^ to 5 x 10^5^ fresh mouse splenocytes were added along with 2.5 μg/ml of ED3 peptide mixtures (a panel of sixteen 15-mer peptides with 9 amino acids overlapping; S1 File) for each serotype or 5 μg/ml concanavalin A (Sigma). After 40 h of incubation, the plates were washed and incubated with a biotinylated antibody against IFN-γ or IL-4 (2 μg/ml) for 2 h at 37°C. After the plates were washed, HRP-conjugated avidin (Research Laboratory Inc.) was added and incubated for 1 h at 37°C. The assays were developed with AEC solution (BD Pharmingen). The reaction was stopped with tap water, and plates were analyzed using an ImmunoSpot reader with ImmunoSpot software, version 5.0.3 (CTL, Cleveland, OH). Data are presented as the number of spot-forming cells (SFC)/10^6^ splenocytes.

### ELISA

ED3-specific IgG titers were determined by ELISA. Briefly, purified recombinant ED3 was coated onto 96-well plates overnight and blocked with 2% FBS in PBS for 2 h at RT. Sera were diluted using 3-fold serial dilutions (starting at 1:100) and added into the wells. Bound IgG was detected with HRP-conjugated goat anti-mouse IgG antibody. After the addition of 3, 3’, 5, 5’-tetramethylbenzidine (TMB), the absorbance was measured with an ELISA reader at 450 nm. ELISA end-point titers were defined as the serum dilution that gave an OD value 2-fold higher than the background. The serum dilution was obtained from the titration curve by interpolation. If the OD value was less than 2-fold of the background at the starting dilution, a titer of 33 was used for calculation.

### Focus reduction neutralization tests (FRNT) and viremia

Sera were diluted using 2-fold serial dilutions (starting at 1:8), and the sera were heat inactivated prior to testing. Monolayers of BHK-21 cells in 24-well plates were inoculated with viruses (DENV-1/Hawaii, DENV-2/16681, DENV-3/H-087, and DENV-4/H241) that had been pre-mixed at 4°C overnight with sera samples to a final volume of 0.5 ml. The virus titers prior to pre-mixing were approximately 20–40 focus-forming units (FFU) per well. Viral adsorption was allowed to proceed for 3 h at 37°C. An overlay medium containing 2% FBS and 0.8% methylcellulose in DMEM was added at the conclusion of adsorption. After 72 h of infection, the cells were fixed for 15 min in 3.7% formaldehyde/PBS, permeabilized with 0.1% Nonidet P40/PBS for 15 min and blocked with 3% BSA/PBS for 30 min. Infected cells were detected with a monoclonal anti-dengue antibody (2H2; American Type Culture Collection, No. HB-114), which reacts with all serotypes of dengue virus. After washing with PBS, antibody-labeled cells were detected with an HRP-conjugated secondary antibody and were visualized using TMB. The FFU were counted, and the neutralizing antibody titer FRNT_50_ was calculated as the endpoint titer that produced a 50% reduction in FFU when compared with virus alone. For calculation purposes, the neutralizing antibody titer was designated as 2^2^ if the neutralizing antibody titer was less than 2^3^.

For the determination of viremia, blood was drawn from mice after challenge and placed in tubes containing the anticoagulant EDTA. Plasma samples were diluted using 10-fold serial dilutions and added onto monolayers of BHK-21 cells in 24-well plates to incubate for 3 h at 37°C for viral adsorption. Infected cells were detected as described above and FFU were counted and represented as FFU/ml.

### Statistical analyses

All statistical analyses were performed using 2-way ANOVA with the Bonferroni post-test (GraphPad Prism), except for the experiments of ELISA and neutralizing antibody, in which Mann-Whitney t-test was used. Differences with a *p* value of less than 0.05 were considered statistically significant.

## Results

### Development of an ED3-based tetravalent DNA vaccine and recombinant subunit vaccine

**Fig 1:**
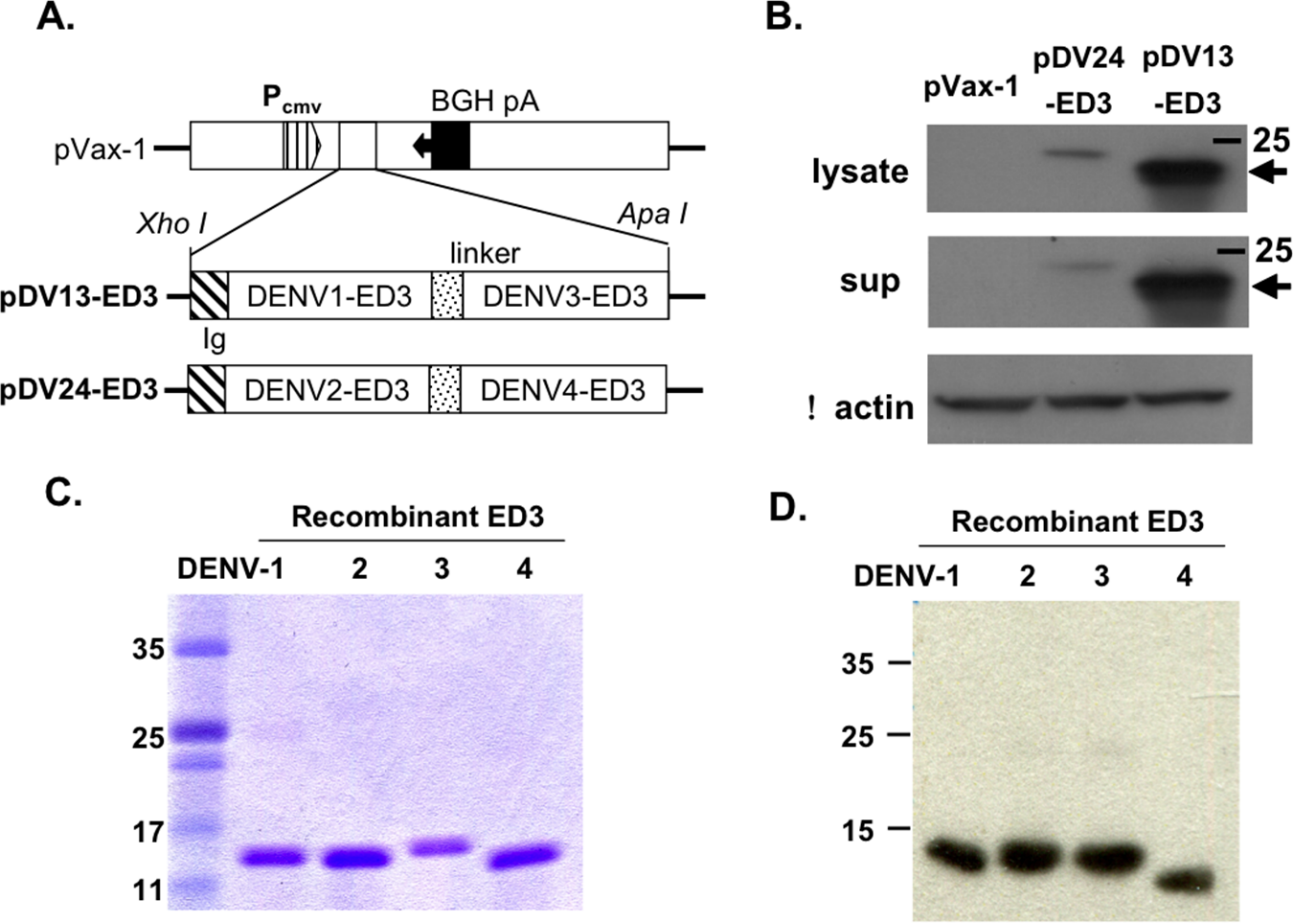
Preparation of a tetravalent ED3-expressing DNA vaccine and a tetravalent recombinant ED3 protein vaccine. (A) Schematic diagram of two ED3-expressing plasmids, pDV13-ED3 and pDV24-ED3, generated by cloning DNA fragments consisting of tandem repeats of ED3 from DENV-1 and 3, and DENV-2 and 4 into the pVax-1 vector between the Xho I and Apa I sites. The positions of the immunoglobulin light chain signal peptide (Ig) or three repeats of GGGGS linker (linker) are also indicated. (B) The presence of bivalent ED3 in the culture supernatants or cell lysates of 293Trex cells transfected with pVax-1, pDV13-ED3 or pDV24-ED3 for 24 h was detected with an ED3-specific monoclonal antibody. The expression of β-actin cellular protein in the cell lysate was used as a control. The recombinant ED3 proteins from DENV-1 to 4 were expressed in *E*. *coli* and purified with an affinity column. The final products were analyzed by SDS-PAGE (C) or Western blotting (D) with hyperimmune sera.

The tetravalent dengue DNA vaccine (pTDV-ED3) was designed as 1:1 mixture of two plasmids encoding the tandem repeats of ED3 from DENV-1 and 3 (pDV13-ED3), and ED3 from DENV-2 and 4 (pDV24-ED3), as shown in Fig 1A. To confirm ED3 protein expression, the supernatant fluids and cell lysates of cultured 293Trex cells transfected with the pVax-1 vector, pDV13-ED3 or pDV24-ED3 were collected for Western blotting. As a control, β-actin in the cell lysates was assayed, and similar signals were clearly observed in all groups (Fig 1B). A single band of approximately 25 kDa was observed in pDV13-ED3 and pDV24-ED3 but not in the pVax-1 control, suggesting that bivalent ED3 was expressed and detected by an anti-ED3 monoclonal antibody (GeneTex). It was also noted that the signal for pDV24-ED3 was relatively weak, possibly due to the binding specificity of the anti-ED3 monoclonal antibody (S1 Fig). The comparable signal intensities in both the culture supernatant and the cell lysate suggested that the bivalent ED3 protein was successfully secreted. In addition to the tetravalent DNA vaccine, a tetravalent recombinant ED3 protein vaccine (rTED3) consisting of the ED3 of four serotypes (10 μg for each plus alum as an adjuvant) was expressed in *E*. *coli* and purified by affinity chromatography as described previously [12]. To verify the purity of the recombinant ED3 protein, the final products were assayed by SDS-PAGE and Western blotting. A single band of 13–14 kDa for the recombinant ED3 of each serotype was observed in a Coomassie blue stained gel (Fig 1C). After blotting with dengue hyperimmune sera, only a single signal with the same size observed in SDS-PAGE was detected for the ED3 of all serotypes (Fig 1D).

### T-cell responses induced by ED3-based tetravalent dengue vaccines

**Fig 2:**
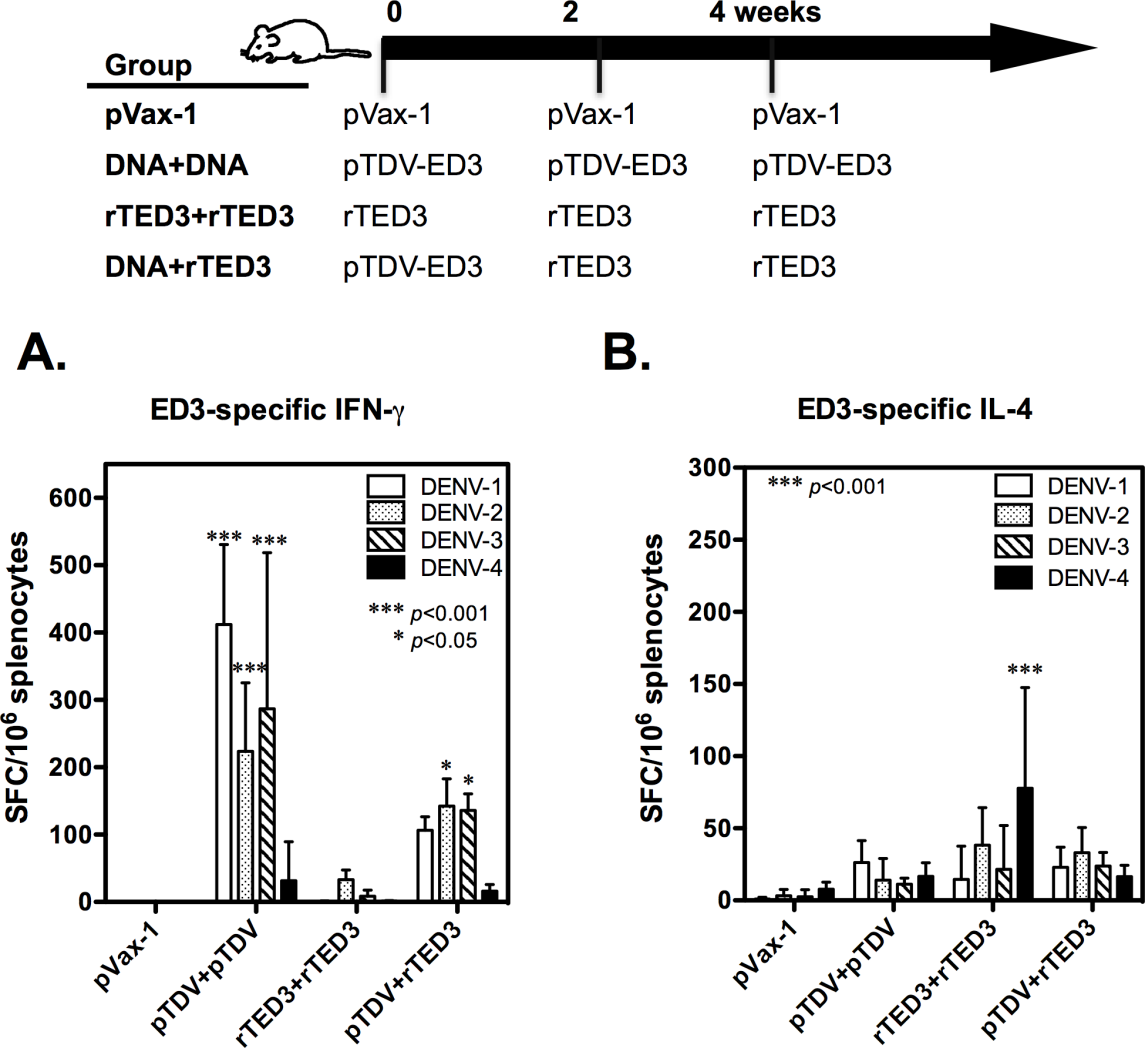
ED3-specific T-cell responses elicited by tetravalent dengue vaccines. Groups of 6- to 8-week-old BALB/c female mice (n = 4) were immunized three times at 2-week intervals with pVax-1, pTDV-ED3 DNA vaccine (pTDV+pTDV), rTED3 protein vaccine (rTED3+rTED3) or prime-boost (pTDV+rTED3) as indicated in the top of figure. One week after last immunization, spleen cells were harvested for the detection of IFN-γ (A) or IL-4 (B) production specific to the ED3 of each serotype by ELISPOT assay. The results are presented with the mean and standard deviation (SD) of spot-forming cells (SFC) per million splenocytes. The significance shown in the graph was determined in comparison to the pVax-1 group, if there is no other indication. The typical results from one out of two independent experiments are presented.

To evaluate the capacity of the tetravalent dengue vaccine to elicit T-cell responses, ED3-specific IFN-γ and IL-4 production in mice receiving three immunizations with the control vector pVax-1, pTDV-ED3, rTED3 vaccine or DNA prime-protein boost strategy using pTDV-ED3 and rTED3 (prime-boost), as shown in the top of Fig 2, were assayed by ELISPOT. No detectable ED3-specific IFN-γ or IL-4 production was observed in pVax-1 immunized mice (Fig 2A). Significant IFN-γ responses to DENV-1, 2 and 3, but not to DENV-4, were induced by pTDV-ED3 (*p*<0.001; n = 4) and the prime-boost (*p*<0.05; n = 4). The numbers of IFN-γ-producing cells in pTDV-ED3-immunized mice were 412±119, 224±101, 287±232 and 32±58 SFC/10^6^ spleen cells in response to DENV-1, 2, 3 and 4, respectively. For prime-boost-immunized mice, the numbers of IFN-γ-producing cells were 107±20, 142±41, 136±25 and 16±10 SFC/10^6^ spleen cells in response to DENV-1, 2, 3 and 4, respectively. In contrast, IL-4 responses were only detected in rTED3 vaccine-immunized mice (15±23, 38±26, 22±30 and 78±70 SFC/10^6^ spleen cells in response to DENV-1 through 4, respectively), and only the IL-4 response to the DENV-4 serotype of ED3 exhibited a significant difference between treatment groups (*p*<0.05, n = 4; Fig 2B).

### Antibody responses induced by tetravalent dengue vaccines

**Fig 3:**
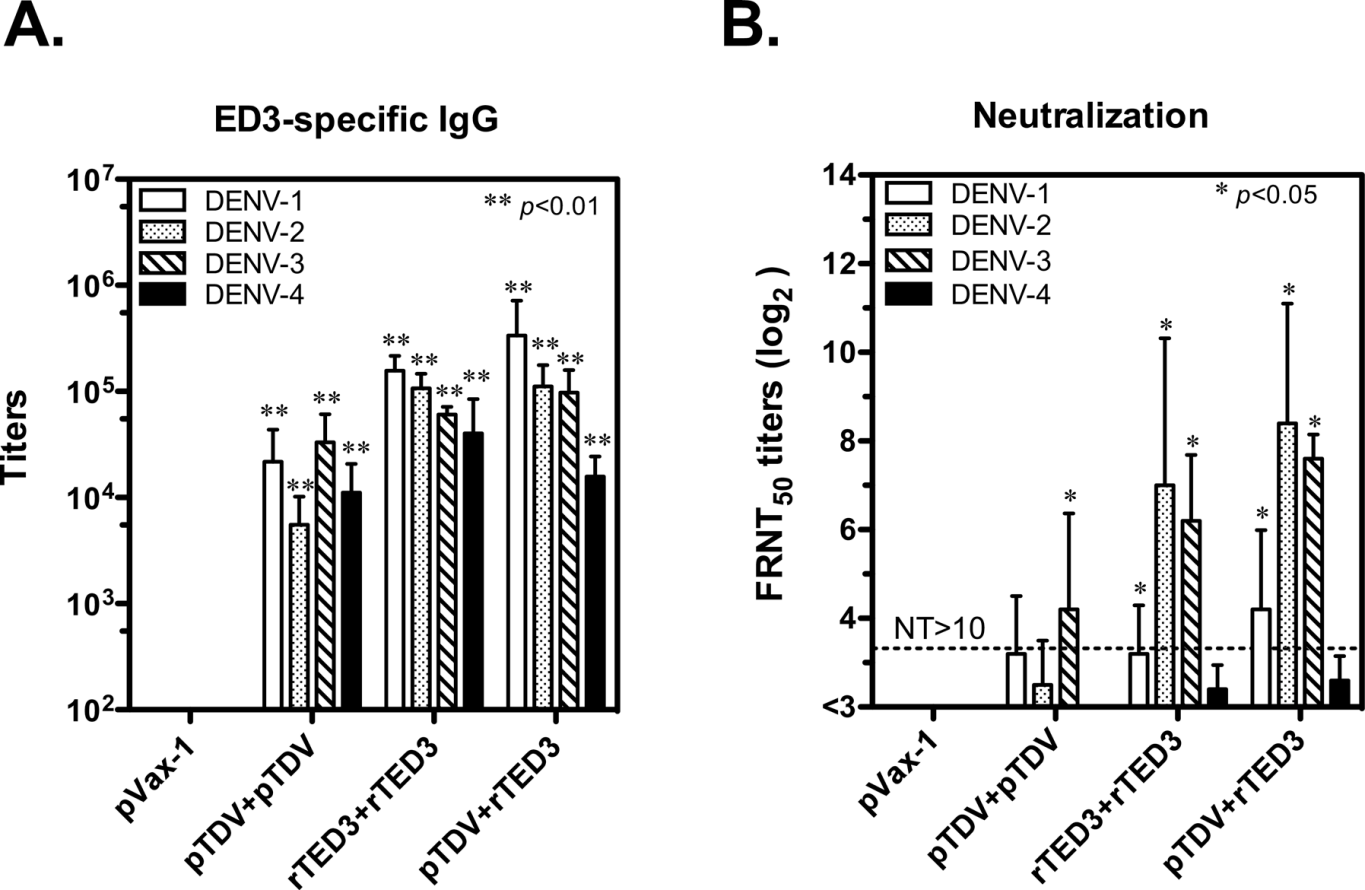
ED3-specific antibody responses elicited by tetravalent dengue vaccines. Groups of 6- to 8-week-old BALB/c female mice (n = 6) were immunized three times at 2-week intervals with pVax-1, pTDV-ED3, rTED3 or prime-boost as the same schedule in Fig 2. The results showing here come from the sera sample collected at week 6 after immunization. (A) The ED3-specific IgG titers assayed by ELISA is presented with the mean and SD. (B) The neutralizing antibody titers against four serotypes of DENV were determined by FRNT, and the endpoint titer leading to ≥50% reduction (FRNT_50_) is shown. Mann-Whitney t-tests were used for statistical analyses, and the significance compared to the pVax-1 group is shown, if nothing else is indicated. The data are representative of two independent experiments with similar results.

One of the major CD4^+^ T-cell functions is to promote antibody production; therefore, we measured the ED3-specific IgG and neutralizing titers against the four serotypes of dengue virus. Only mice immunized with dengue vaccines, and not the control vector, developed a significant ED3-specific IgG response to the four serotypes of ED3 (*p*<0.01 according to Mann-Whitney t-test; n = 5; Fig 3A). Mice immunized with the rTED3 vaccine and the prime-boost elicited higher but not significant IgG titers than pTDV-ED3-immunized mice. Similar to the IgG responses, higher neutralizing titers were observed in mice receiving the rTED3 vaccine and the prime-boost immunization than those observed in pTDV-ED3-immunized mice and showed a significant difference in neutralizing titers to DENV-1, 2 and 3, comparing to the pVax-1 control (*p*<0.05 according to Mann-Whitney t-test; n = 5; Fig 3B). Regarding to serotype-specific antibody responses, the comparable IgG titers for four serotypes were detected in tetravalent dengue vaccines-immunized mice. In contrast, neutralizing antibody responses showed a big serotype-dependent difference and neutralizing titers for DENV-2 and 3 were highest, followed by those for DENV-1. DENV-4 neutralizing titers were the lowest and failed to exceed the value of 10, which is thought to be protective in infants [27].

### ED3-specific CD4-restricted T-cell epitopes identified in dengue virus DNA-immunized mice

**Fig 4:**
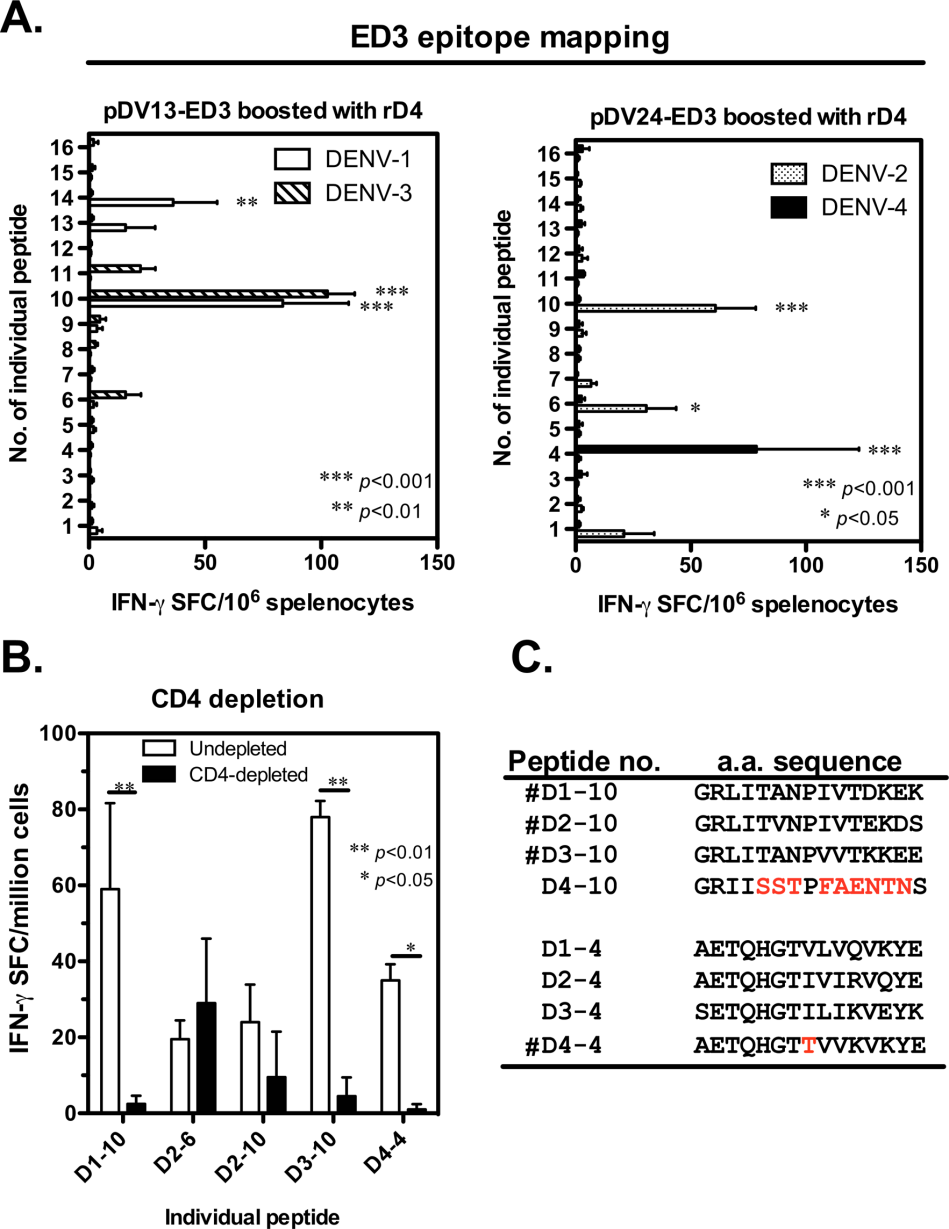
ED3-specific T-cell epitope mapping. Mice (n = 4 per group) were immunized with either pDV13-ED3 or pDV24-ED3 and boosted with recombinant DENV-4 ED3 (rD4) with alum 2 weeks later. (A) Spleen cells were harvested 1 week after the boost, and T-cell epitopes were mapped using IFN-γ ELISPOT. The mean and SD of IFN-γ SFC per million cells responding to sixteen individual peptides for each serotype ED3 are shown. The significance shown in the graph was determined in comparison to other individual peptides. (B) Part of spleen cells were treated with anti-CD4 antibody conjugated with magnetic beads (MACs) for CD4^+^ cells depletion or were left untreated to analyze the CD4 dependence of specific IFN-γ response by ELISPOT. The mean and SD of IFN-γ SFC per million cells with or without CD4-depletion are indicated. (C) The amino acid sequences of ED3 peptide number 10 (E_349-363_) and 4 (E_313-327_) from DENV-1 to 4 are listed.

To understand whether the poor IFN-γ response to DENV-4 after tetravalent vaccination was due to a lack of T-cell epitopes or not, we further analyzed the T-cell epitopes located in the ED3 regions. Mice were immunized with pDV13-ED3 or pDV24-ED3 and boosted with recombinant DENV-4 ED3 protein to induce a stronger DENV-4 specific T-cell response. A panel of sixteen overlapping individual peptides for each serotype ED3 was used to screen for specific IFN-γ production (S1 File). Two peptides (D1-10 and D1-14) from DENV-1 and one peptide (D3-10) from DENV-3 were found to induce significant IFN-γ responses than other peptides in pDV13-ED3-immunized mice (*p*<0.01; n = 4; Fig 4A). In pDV24-ED3-immunized mice, two peptides (D2-6 and D2-10) from DENV-2 and one peptide (D4-4) from DENV-4 were identified to induce significant IFN-γ responses than the other peptides (*p*<0.05; n = 4). Interestingly, an epitope region (D1-10, D2-10 and D3-10) located at E_349-363_ was shared by DENV-1-, 2- and 3-specific T cells, whereas DENV-4-specific T cells recognized a different epitope located at E_313-327_. To test whether the specific IFN-γ response was CD4^+^T-cell dependent, CD4-depleted and non-depleted spleen cells were used to assay IFN-γ production after stimulation by selected peptides. As a control, the peptide D2-6 (E_325-339_; QYEGDGSPCKIPFEI) containing an L^d^-restricted CD8^+^ T-cell epitope (underlined) [28] was also included. Specific IFN-γ responses were significantly reduced in CD4-depleted spleen cells stimulated with D1-10, D3-10 and D4-4 (*p*<0.05; n = 2; Fig 4B). The response to D2-10 was also decreased in CD4-depleted cells but was not significantly different compared to the response observed in non-depleted cells. In contrast, the IFN-γ response to D2-6 stimulation was unchanged regardless of CD4 depletion. The amino acid sequences for E_349-363_ and E_313-327_ of the four serotypes are summarized in Fig 4C. The homology of the amino acid sequence for E_349-363_ ranges from 67 to 80% when comparing DENV-1, 2 and 3 but drops to <33% when comparing DENV-4 and the other three serotypes. In contrast, there is high homology for the amino acid sequence of E_313-327_ when comparing DENV-4 and the other three serotypes (80%, 73% and 60% identity for DENV-1, 2 and 3, respectively), but it appears that T cells induced by the tetravalent DNA vaccine are capable of distinguishing the difference and reacting specifically to E_313-327_ of DENV-4.

### Changes in epitope-specific T cells following multiple boosts in tetravalent vaccine-immunized mice

**Fig 5:**
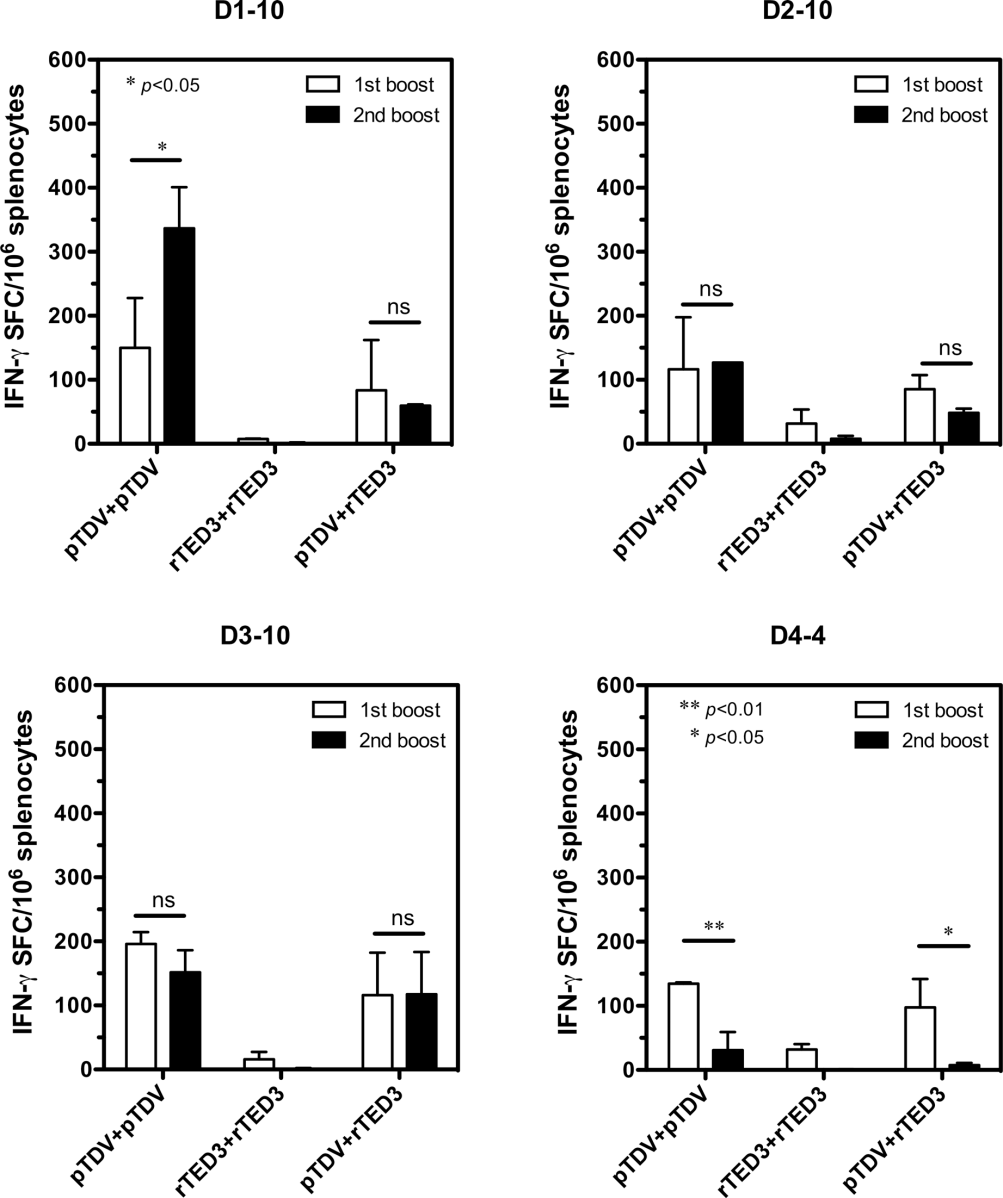
The time-course changes in CD4^+^ T-cell epitope-specific responses after tetravalent dengue vaccination. Groups of 6- to 8-week-old BALB/c female mice (n = 4) were immunized three times at 2-week intervals with pTDV-ED3, rTED3 or prime-boost as indicated in Fig 2. Spleen cells from two mice per group were harvested one week after either the 1^st^ or 2^nd^ boost for the detection of IFN-γ production by ELISPOT. The mean and SD of SFC per million splenocytes responding to stimulation with the indicated individual peptides are shown.

To understand why the D4-4 peptide-specific T cells responded poorly to tetravalent vaccines, we assayed the time course change of IFN-γ responses in immunodominant epitope-specific T cells. Groups of mice (n = 4) immunized with pTDV-ED3, rTED3 vaccine or prime-boost as described previously were sacrificed after 1^st^ or 2^nd^ boost for the detection of epitope-specific T-cell responses. As in the previous stimulation with pooled peptides, only pTDV-ED3 and prime-boost immunizations demonstrated IFN-γ production in response to the stimulation with the individual peptides D1-10, D2-10, D3-10 and D4-4 (Fig 5). Surprisingly, in contrast to the increased or unchanged high levels of IFN-γ responses specific to D1-10, D2-10 and D3-10, the D4-4-specific IFN-γ response was significantly reduced in both pTDV-ED3- (*p*<0.01; n = 2) and prime-boost-immunized mice (*p*<0.05; n = 2) following the second boost. It is apparent that immunodominance changed and was biased to the T-cell epitope shared by DENV-1 to 3.

### Protection against DENV-4 challenge by tetravalent dengue vaccination

**Fig 6:**
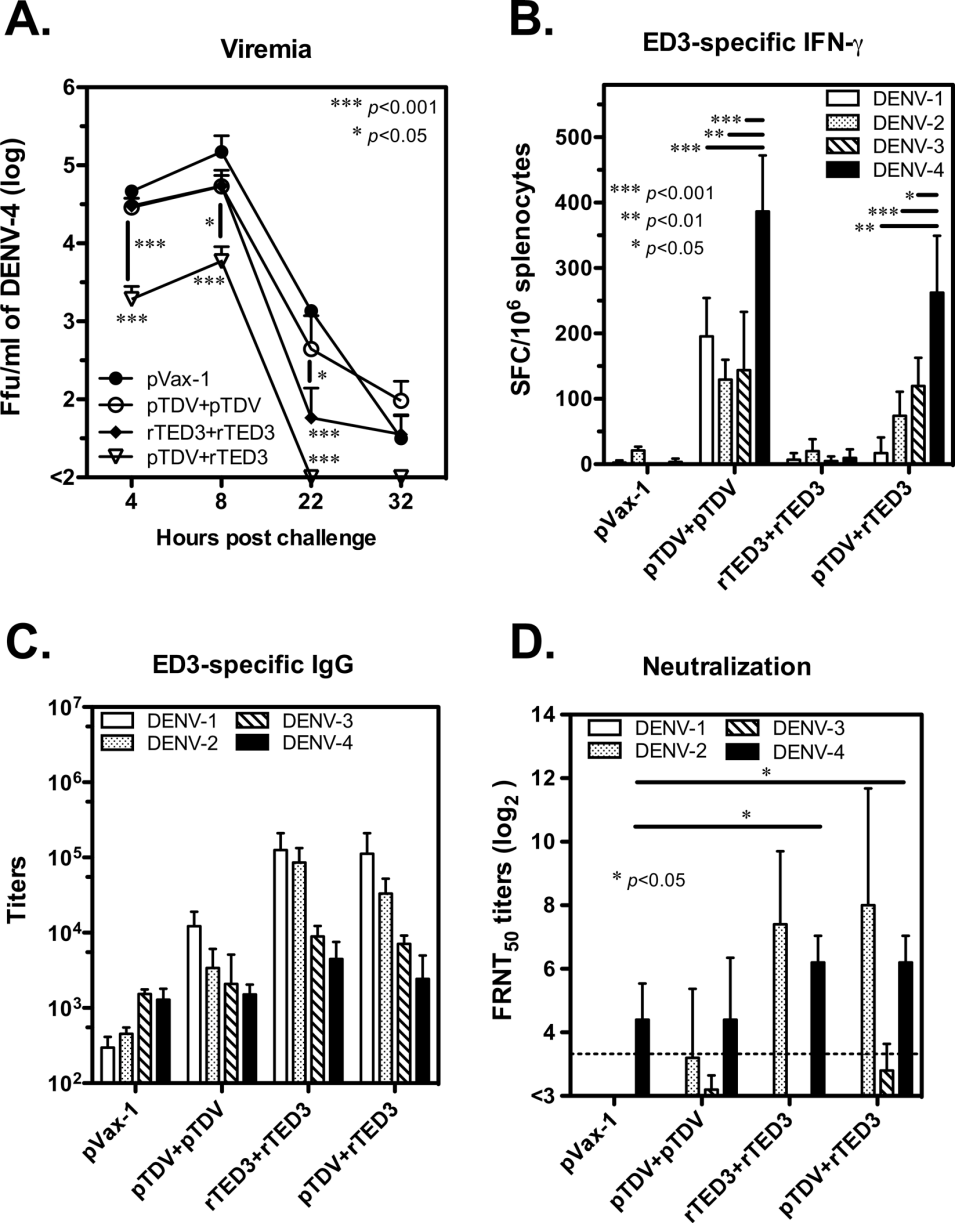
Protection, IFN-γ responses and antibody titers in tetravalent dengue vaccine-immunized mice after DENV-4 challenge. Groups of 6- to 8-week-old BALB/c female mice (n = 6, except n = 4 for pVax-1) were immunized three times at 2-week intervals with pVax-1, pTDV-ED3, rTED3 or prime-boost as the same schedule in Fig 2. Four weeks after the last immunization, mice were challenged with the intraperitoneal injection of 5 x 10^7^ DENV-4 infected K562 cells. (A) Plasma viremia titers from individual mice were determined by viremia assay and are represented as the mean and SD. The significance shown in the graph was determined in comparison to the pVax-1 group, if nothing else is indicated. (B) Spleen cells were harvested for the detection of the ED3-specific IFN-γ production by ELISPOT at 1 month post-challenge. (C and D) The sera collected at 1 month post-challenge were used for the detection of neutralizing titers in an FRNT assay (C) or ED3-specific IgG titers by ELISA (D).

To evaluate the effects of the immunodominance change on protection against DENV-4 infection, mice immunized with the pVax-1, pTDV-ED3, rTED3 or prime-boost vaccines were challenged with DENV-4. In the pVax-1 control, the DENV-4 virus was detected in circulating blood 4 h after challenge with a mean viremia titer of 5 x 10^4^ FFU/ml, reaching a peak of 2 x 10^5^ FFU/ml at 8 h post-challenge and decreasing below the detection limit (10^2^ FFU/ml) at 32 h after challenge (Fig 6A). No significant difference in viremia titer was observed between pTDV-ED3-immunzed mice and the pVax-1 control during the course of infection, suggesting that the pTDV-ED3-elicited immune response fails to contribute to protection against dengue infection. For the rTED3 vaccine, the initial and peak viral loads were similar to those for pTDV-ED3, but viral loads quickly and significantly decreased after the peak (*p*<0.001 and *p*<0.05 comparing rTED3 to pVax-1 and pTDV-ED3, respectively; n = 6 except n = 4 for pVax-1). The lowest initial viral load and the peak titer were observed in prime-boost-immunized mice and demonstrated significant differences for the pTDV-ED3 and rTED3 vaccines (*p*<0.05) and the pVax-1 control (*p*<0.001). Moreover, DENV-4 was cleared from the circulation 22 h after challenge in prime-boost-immunized mice, which was faster than the clearance observed in the other groups. To determine whether DENV-4-specific immunity was recalled by the challenge, we analyzed ED3-specific IFN-γ production and antibody responses 1 month after challenge. DENV-4 ED3-specific IFN-γ production was not observed for the rTED3 vaccine and the pVax-1 control, suggesting that ED3 is not a major T-cell response area after virus infection (Fig 6B). However, the DENV-4 specific IFN-γ response in pTDV-ED3- and prime-boost-immunized mice was significantly higher than other serotypes, suggesting that DENV-4 ED3-specific immunity was recalled. Regarding to the antibody responses after challenge, DENV-4 specific neutralizing titers increased in all groups and were even significantly higher for both rTED3-and prime-boost-immunized mice than the pVax-1 control (*p*<0.05 according to Mann-Whitney t-test; Fig 6C). In contrast, DENV-4 specific IgG titers unchanged after challenge and remained lower than other serotypes in the tetravalent dengue vaccine groups but not in the pVax-1 control, for which a higher DENV-4 specific IgG response was observed (Fig 6D).

## Discussion

Successful development of a safe and efficient tetravalent dengue vaccine will be aided by the systematically analysis of CD4^+^ T-cell responses. Previous studies to identify CD4^+^ T-cell responses were performed on the subjects infected with dengue, mostly for DENV-2 and analyzed at a genome-wide scale. These results are valuable for understanding the virus infection-induced T-cell responses but may not reflect the T-cell immunity elicited by vaccination, particularly for tetravalent vaccines, which contain highly homologous antigens from viruses of four serotypes. To address this issue, we focus on the ED3, which is the major target site for neutralizing antibodies, to characterize the ED3-specific CD4^+^ T-cell responses elicited by tetravalent dengue vaccines. Two vaccination approaches, comprising DNA immunization and recombinant protein with alum adjuvant were used for the induction of potent Th1 and Th2 responses. The different patterns of IFN-γ and IL-4 responses induced against four serotypes suggest that ED3-specific CD4^+^ T-cell responses are heterogeneous and serotype-dependent. In addition, the serotype-specificity of CD4^+^ T-cell responses was further confirmed by the monovalent ED3-based DNA vaccine (S2 Fig). Our data are in agreement with the finding that T cells derived from mice immunized with a DNA vaccine expressing full pre-membrane and envelope proteins only responded to homologous virus antigen [29]. Considering that the amino acid homologies between the ED3 of the four serotypes ranged from 71 to 50% (with the lowest identity observed for DENV-4) [24], the serotype-dependent ED3-specific T-cell responses are not surprising. The specificity of T-cell responses is very important because cross-reactive T-cell responses have been proposed to be involved in the pathogenesis of dengue infection [30].

It is of particular interest to identify the immunodominant T-cell epitopes in ED3 for vaccine development as well as for the study of dengue pathogenesis. Surprisingly, our epitope mapping results identified two CD4^+^ T-cell epitopes; one recognized by T cells specific to DENV-1 to 3 is located at E_349-363_, and the other, which is located at E_313-327_, is recognized by DENV-4-specific T cells. Both epitope-specific CD4^+^ T cells were induced with early priming; however, the decrease in DENV-4- but not other serotype-specific IFN-γ responses with later boosting suggests that the DENV-4 epitope became sub-dominant to the other serotypes. The immunodominance between different T cell epitopes is a normal immunological phenomenon (or called immunodomination) caused by immunodominant T cells suppressing responses to less-dominant epitopes through MHC-peptide complex competition and other mechanisms [31–33]. This phenomenon has also been observed for several types of viral infections and is associated with protective immunity [34–37]. To our knowledge, this is the first report of differential immunodominance for different serotype antigens causing imbalanced serotype-specific IFN-γ responses during tetravalent vaccination. To understand the influence of decreased DENV-4-specific IFN-γ responses in protection against dengue infection, DENV-4 was used in a challenge test. The fact that the recalled DENV-4-specific IFN-γ responses were observed in DNA vaccine-primed mice after challenge suggests that DENV-4-specific T cell precursors still exist 8 weeks after vaccination and respond normally to homologous antigen stimulation. However, determining the length of time that DENV-4-specific T cell precursors can be maintained and their contribution to protection requires further investigation.

The protective mechanisms that are engaged against dengue infection are unclear due to the lack of an animal model that is relevant to humans; however, neutralizing antibodies and IFN-γ production have been reported to be involved. When comparing the two tetravalent dengue vaccines that we used, immune responses induced by the DNA vaccine or protein vaccine were dominated by either IFN-γ production or neutralizing antibodies. Protein-immunized mice but not DNA-immunized mice demonstrated lower viremia than the control group suggesting that neutralizing antibodies are more important than IFN-γ production to against DENV infection. Interestingly, the prime-boost-immunized mice that generated both IFN-γ production and neutralizing antibodies also had the lowest viremia compared to other groups, suggesting a synergetic effect between IFN-γ production and neutralizing antibodies for viral clearance. Recently, it has been reported that cytotoxic CD4^+^ T cells play a role in protective immunity against dengue infection [38]. We cannot rule out the possibility that ED3-specific CD4^+^ T cells can kill the virus-infected cells directly; however, the challenge model we used cannot address this issue due to the mismatched MHC and an interferon deficient AG129 mouse model is an alternative for the evaluation of cytotoxic T cells in protective immunity, as previous described [39].

Heterologous vaccination with a prime-boost strategy consisting of different vaccines has been shown to improve immunogenicity [40, 41]. Our prime-boost vaccination also confirmed this improvement by eliciting both high levels of IFN-γ production and neutralizing antibodies, which are thought to play an important role against dengue infection. Therefore, the results of our prime-boost vaccination highlight its potential as a tetravalent dengue vaccine candidate. Given that DENV-4-specific IFN-γ responses were undetectable in the control and protein-vaccinated mice but were the strongest of all serotype-specific IFN-γ responses in DNA- or prime-boost-immunized mice after DENV-4 challenge, it appears that ED3 is not a major target for virus-induced immune responses. However, vaccine-elicited ED3-specific T cells can respond strongly to DENV infection and contribute to protection.

## Conclusions

In summary, we demonstrate here that the ED3-specific CD4^+^ T-cell responses elicited by tetravalent vaccines are serotype-specific and are affected by immunodominance change between T-cell epitopes. Furthermore, induction of IFN-γ responses and neutralizing antibodies with prime-boost heterologous vaccination results in more efficient virus clearance than is observed after DNA or protein homologous vaccination. This information should be valuable for the future development of safe and efficacious tetravalent dengue vaccines.

## Supporting Information

S1 Fig The reactivity of anti-dengue monoclonal antibody. Purified recombinant ED3 of the four serotypes (0.5 μg each) were loaded into the wells of a 4–20% gradient SDS-PAGE for electrophoresis, transferred and blotting with an anti-dengue ED3 monoclonal antibody (GeneTex).
(JPG)

S2 Fig Serotype-specificity of T-cell responses induced by the tetravalent dengue vaccines. Mice were immunized two times at 2-week intervals with 100 μg of monovalent pDV-ED3, pDV2-ED3, pDV3-ED3 or pDV4-ED3 plasmid by im injection. Spleen cells were removed 3 weeks after immunization, and assayed for IFN-γ production by ELISPOT. The IFN-γ production in response to the stimulation with ED3 of four serotypes was shown.
(JPG)

S3 Fig IgG isotype pattern od ED3-specific antibody responses induced by the tetravalent dengue vaccines. Mice were immunized three times with pTDV-ED3, rTED3 or prime-boost as the same immunization schedule and dosage in Fig 2, and the reciprocal titers of ED3-sepcifc IgG1 and IgG2a were determined by ELISA described previously, except the HRP-conjugated anti-mouse IgG antibody was replaced with biotinated anti-mouse IgG1 or IgG2a and avidin-HRP (all BD Biosciences). The mean and SD of IgG1/2a ratio from each mouse (n = 4 or 5) were shown.
(JPG)

S4 Fig D4-4 specific T cells were boosted after DENV-4 challenge. Mice were immunized three times with pTDV-ED3, rTED3 or prime-boost and challenge with DENV-4 infected K562 cells as the same in Fig 6. Spleen cells were harvested 4 weeks later for detection of IFN-γ production in response to the stimulation with either DENV-4 pooled peptides or D4-4 individual peptide. The mean and SD of spot forming cells per million spleen cells were shown (n = 2). More than 50% of DENV-4 specific IFN-γ producing cells in pTDV-ED3 or prime-boost immunized mice were targeted to D4-4.
(JPG)

S1 File ED3 (E_295-397_) peptides used for T cell stimulation. (DOC)
