# Supplementary figures and images for: The Immunodominance Change and Protection of CD4+ T-Cell Responses Elicited by an Envelope Protein Domain III-Based Tetravalent Dengue Vaccine in Mice
Source: PLoS One. 2015 Dec 29;10(12):e0145717. doi: 10.1371/journal.pone.0145717 (PMC4695087; doi:10.1371/journal.pone.0145717)

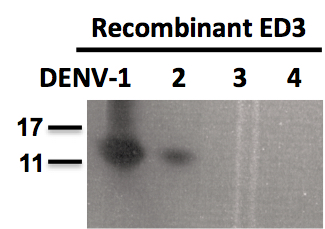

Supplement: S1 Fig — Purified recombinant ED3 of the four serotypes (0.5 μg each) were loaded into the wells of a 4–20% gradient SDS-PAGE for electrophoresis, transferred and blotting with an anti-dengue ED3 monoclonal antibody (GeneTex). (JPG) [file pone.0145717.s001.jpg]

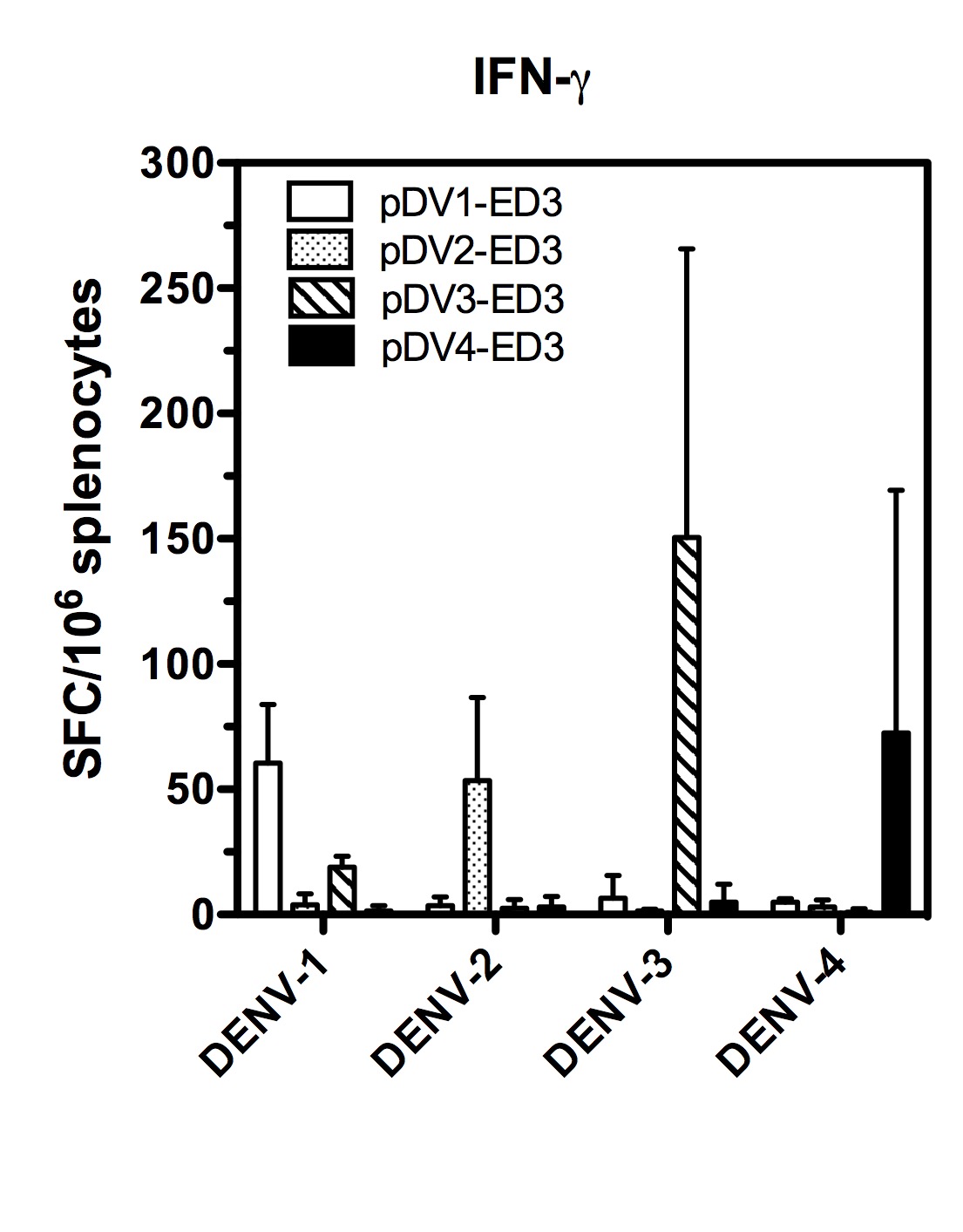

Supplement: S2 Fig — Mice were immunized two times at 2-week intervals with 100 μg of monovalent pDV-ED3, pDV2-ED3, pDV3-ED3 or pDV4-ED3 plasmid by im injection. Spleen cells were removed 3 weeks after immunization, and assayed for IFN-γ production by ELISPOT. The IFN-γ production in response to the stimulation with ED3 of four serotypes was shown. (JPG) [file pone.0145717.s002.jpg]

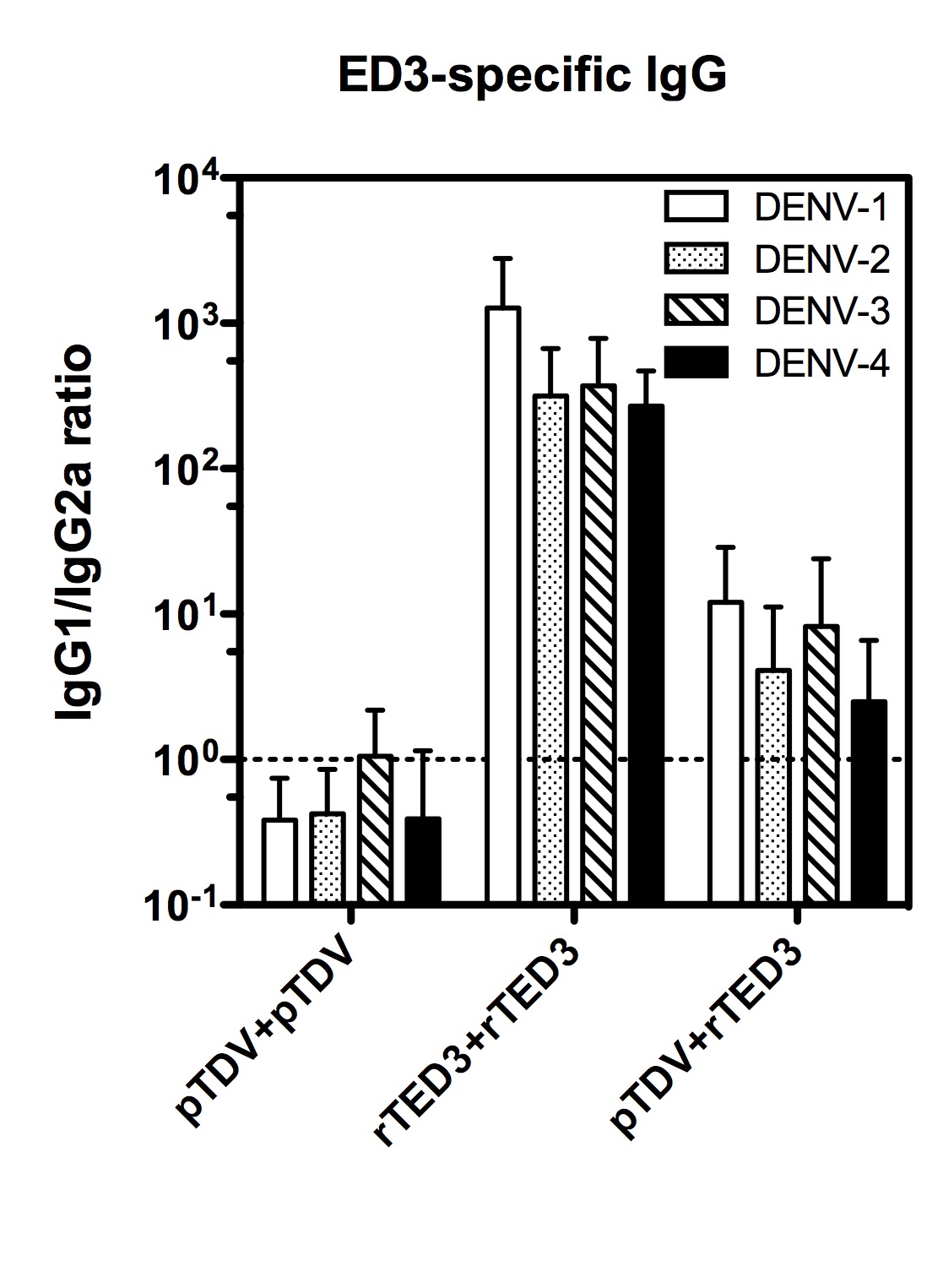

Supplement: S3 Fig — Mice were immunized three times with pTDV-ED3, rTED3 or prime-boost as the same immunization schedule and dosage in Fig 2, and the reciprocal titers of ED3-sepcifc IgG1 and IgG2a were determined by ELISA described previously, except the HRP-conjugated anti-mouse IgG antibody was replaced with biotinated anti-mouse IgG1 or IgG2a and avidin-HRP (all BD Biosciences). The mean and SD of IgG1/2a ratio from each mouse (n = 4 or 5) were shown. (JPG) [file pone.0145717.s003.jpg]

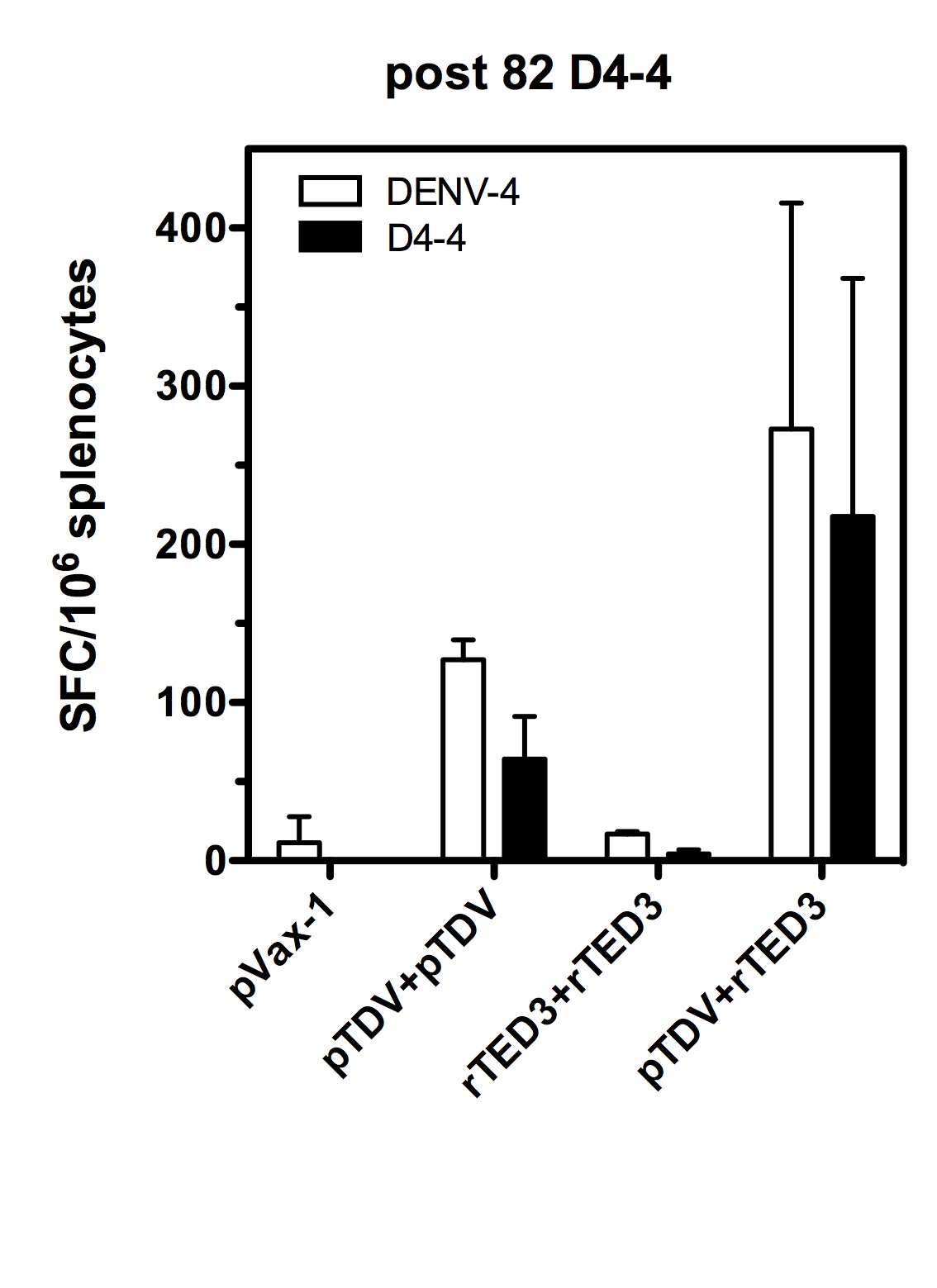

Supplement: S4 Fig — Mice were immunized three times with pTDV-ED3, rTED3 or prime-boost and challenge with DENV-4 infected K562 cells as the same in Fig 6. Spleen cells were harvested 4 weeks later for detection of IFN-γ production in response to the stimulation with either DENV-4 pooled peptides or D4-4 individual peptide. The mean and SD of spot forming cells per million spleen cells were shown (n = 2). More than 50% of DENV-4 specific IFN-γ producing cells in pTDV-ED3 or prime-boost immunized mice were targeted to D4-4. (JPG) [file pone.0145717.s004.jpg]
