# Supplementary material for: The Immunodominance Change and Protection of CD4+ T-Cell Responses Elicited by an Envelope Protein Domain III-Based Tetravalent Dengue Vaccine in Mice
Source: PLoS One. 2015 Dec 29;10(12):e0145717. doi: 10.1371/journal.pone.0145717 (PMC4695087; doi:10.1371/journal.pone.0145717)
Supplement: S1 File — (DOC) [file pone.0145717.s005.doc]

**S1 File. ED3 (E_295-397_**) peptides used for T cell stimulation.

| DENV-1 consensus ED3 | |  | | DENV-2 consensus ED3 | | |  | |
| --- | --- | --- | --- | --- | --- | --- | --- | --- |
| **Name** | **A.A. sequence** | |  | **Name** | | **A.A. sequence** | |  |
| D1-1 | **KGMSYVMCTGSFKLE** | |  | D2-1 | | **KGMSYSMCTGKFKVV** | |  |
| D1-2 | **MCTGSFKLEKEVAET** | |  | D2-2 | | **MCTGKFKVVKEIAET** | |  |
| D1-3 | **KLEKEVAETQHGTVL** | |  | D2-3 | | **KVVKEIAETQHGTIV** | |  |
| D1-4 | **AETQHGTVLVQVKYE** | |  | D2-4 | | **AETQHGTIVIRVQYE** | |  |
| D1-5 | **TVLVQVKYEGTDAPC** | |  | D2-5 | | **TIVIRVQYEGDGSPC** | |  |
| D1-6 | **KYEGTDAPCKIPFSS** | |  | D2-6 | | **QYEGDGSPCKIPFEI** | |  |
| D1-7 | **APCKIPFSSQDEKGV** | |  | D2-7 | | **SPCKIPFEIMDLEKR** | |  |
| D1-8 | **FSSQDEKGVTQNGRL** | |  | D2-8 | | **FEIMDLEKRHVLGRL** | |  |
| D1-9 | **KGVTQNGRLITANPI** | |  | D2-9 | | **EKRHVLGRLITVNPI** | |  |
| D1-10 | **GRLITANPIVTDKEK** | |  | D2-10 | | **GRLITVNPIVTEKDS** | |  |
| D1-11 | **NPIVTDKEKPVNIEA** | |  | D2-11 | | **NPIVTEKDSPVNIEA** | |  |
| D1-12 | **KEKPVNIEAEPPFGE** | |  | D2-12 | | **KDSPVNIEAEPPFGD** | |  |
| D1-13 | **IEAEPPFGESYIVVG** | |  | D2-13 | | **IEAEPPFGDSYIIIG** | |  |
| D1-14 | **FGESYIVVGAGEKAL** | |  | D2-14 | | **FGDSYIIIGVEPGQL** | |  |
| D1-15 | **VVGAGEKALKLSWFK** | |  | D2-15 | | **IIGVEPGQLKLNWFK** | |  |
| D1-16 | **KALKLSWFKKGSS** | |  | D2-16 | | **GQLKLNWFKKGSS** | |  |
|  |  | |  |  |  | |  | |
| DENV-3 consensus ED3 | | |  | DENV-4 consensus ED3 | | |  | |
| **Name** | **A.A. sequence** | |  | **Name** | | **A.A. sequence** | |  |
| D3-1 | **KGMSYAMCLNTFVLK** | |  | D4-1 | | **KGMSYTMCSGKFSID** | |  |
| D3-2 | **MCLNTFVLKKEVSET** | |  | D4-2 | | **MCSGKFSIDKEMAET** | |  |
| D3-3 | **VLKKEVSETQHGTIL** | |  | D4-3 | | **SIDKEMAETQHGTTV** | |  |
| D3-4 | **SETQHGTILIKVEYK** | |  | D4-4 | | **AETQHGTTVVKVKYE** | |  |
| D3-5 | **TILIKVEYKGEDAPC** | |  | D4-5 | | **TTVVKVKYEGAGAPC** | |  |
| D3-6 | **EYKGEDAPCKIPFST** | |  | D4-6 | | **KYEGAGAPCKVPIEI** | |  |
| D3-7 | **APCKIPFSTEDGQGK** | |  | D4-7 | | **APCKVPIEIRDVNKE** | |  |
| D3-8 | **FSTEDGQGKAHNGRL** | |  | D4-8 | | **IEIRDVNKEKVVGRI** | |  |
| D3-9 | **QGKAHNGRLITANPV** | |  | D4-9 | | **NKEKVVGRIISSTPF** | |  |
| D3-10 | **GRLITANPVVTKKEE** | |  | D4-10 | | **GRIISSTPFAENTNS** | |  |
| D3-11 | **NPVVTKKEEPVNIEA** | |  | D4-11 | | **TPFAENTNSVTNIEL** | |  |
| D3-12 | **KEEPVNIEAEPPFGE** | |  | D4-12 | | **TNSVTNIELEPPFGD** | |  |
| D3-13 | **IEAEPPFGESNIVIG** | |  | D4-13 | | **IELEPPFGDSYIVIG** | |  |
| D3-14 | **FGESNIVIGIGDKAL** | |  | D4-14 | | **FGDSYIVIGVGDSAL** | |  |
| D3-15 | **VIGIGDKALKINWYK** | |  | D4-15 | | **VIGVGDSALTLHWFR** | |  |
| D3-16 | **KALKINWYKKGSS** | |  | D4-16 | | **SALTLHWFRKGSS** | |  |
